# Nucleoside-Analog Reverse-Transcriptase Inhibitors (NRTIs) Against Multiple Sclerosis: Comprehensive Review on a Possible Novel Therapeutic Approach

**DOI:** 10.3390/neurolint18050089

**Published:** 2026-05-12

**Authors:** Alfonso Martinisi, Paolo Paganetti

**Affiliations:** 1 Faculty of Economics, Università della Svizzera Italiana (USI), 6900 Lugano, Switzerland; 2 Tivenix SA, 6900 Lugano, Switzerland; ppaganetti@tivenix.com

**Keywords:** multiple sclerosis, NRTI, HERV, antiretrovirals, remyelination

## Abstract

To this day, the etiology of multiple sclerosis has yet to be fully comprehended by the scientific community. However, the knowledge on mechanisms leading to the development of this neurodegenerative autoimmune disorder increases daily, along with the development of new disease-modifying treatments. A correlation between Epstein–Barr Virus infection and the disease incidence has recently shed light on possible innovative antiviral therapies. Here, we review the literature on Human Endogenous Retroviral sequences as emerging actors for the impairment of remyelination as a major challenge in disease progression. Our primary focus is the HERV-W envelope protein, which has been found at elevated levels in individuals affected by this condition and is suggested here as a potential therapeutic target. We then continue analyzing the clinical cases where antiretroviral drugs have been tested to treat multiple sclerosis patients and, from successes and failures, we finally narrow down our therapeutic hypothesis to the administration of Nucleoside-analog Reverse Transcriptase Inhibitors to target the HERV-W envelope protein, possibly leading to remyelination and significantly improving the condition of those affected by the disease. The main purpose of this review is to present a rationale for the therapeutic potential of this drug class and offer a new perspective for therapeutic options against multiple sclerosis.

## 1. Multiple Sclerosis: Characteristics of the Disease, Main Therapeutic Approaches and Unanswered Questions

Multiple sclerosis (MS) is an autoimmune disorder that develops as a chronic inflammatory and neurodegenerative disease of the central nervous system (CNS) [1]. It is one of the most frequent causes of neurological disability in young adults, displaying a vast array of symptoms such as fatigue, visual impairments, progressive motor disability and various degrees of cognitive dysfunction, among others [2], and it has a significant social impact on affected patients [3]. Although knowledge about various elements of the disease is growing, some aspects are still not fully understood [4]. Its autoimmune characteristics are an example of that, as they have been linked historically with a T-cell-mediated response [5], but recent successful therapies targeting B-cells [6] suggest a more complex cellular involvement [5]. MS is known to deteriorate the myelin sheath of neurons produced by oligodendrocytes in different, patient-specific regions of the brain, cranial nerves, brain stem, cerebellum, and spinal cord [7]. This degeneration affects neuronal function and may lead to neuronal loss, eventually causing the typical symptoms associated with MS. Nevertheless, numerous neurological challenges remain not fully understood. For instance, the precise mechanisms that cause inefficient remyelination of nervous system lesions remain unclear [8] due to the complex molecular interplay that allows for the process of remyelination and the variable extent thereof in MS patients [9]. Although our understanding of the causes of MS is still limited, noteworthy advancements have been achieved with injectable and oral medications that fight symptoms or slow down disease progression [10]. As a result, major disabilities are now largely prevented, and the life expectancy of patients diagnosed with MS is only marginally reduced. Since the introduction of interferon (IFN) β-1b in 1993 as the first therapeutic drug for the disease [10], a plethora of drugs have been approved for treating MS, almost entirely dedicated to the relapse-remitting MS form (RRMS) [11], but not only that, treatments showing modest but clinically meaningful effects have been approved for the management of progressive multiple sclerosis, such as Ocrelizumab for primary progressive MS (PPMS) and Siponimod for secondary progressive MS (SPMS) [12]. Notwithstanding these remarkable improvements, MS remains a not-fully met medical need, considering that the available therapies only slow down the disease, that no definitive cure exists [13], and that they show limited efficacy for PPMS and SPMS [14].

The neuroprotective and immunomodulating mechanisms of the approved drugs are not entirely clear. The first approved drug against MS, injectable IFN-β, is mainly thought to cause a shift in the regulation of the cellular immune response, decreasing the threat of autoimmune reactions against the nerve cells [15], but it may also act at a genetic level by correcting gene dysregulation in MS [16]. Also, the mechanism of action of the increasingly relevant anti-CD20 antibodies (such as the aforementioned Ocrelizumab), although more straightforward, is not completely understood and some of the consequences of the treatment, such as the B-cell depletion and the kinetics of this process, still evade full comprehension [17]. The first oral treatment against MS, Fingolimod, induces an internalization of Sphingosine-1-Phosphate-1 (S1P1) receptors, reducing the penetration of lymphocytes in the CNS and, consequently, MS-related inflammation [18]. However, its selectivity for the S1P1 receptor had to be improved to avoid safety issues such as bradycardia, causing a wave of second-generation S1P1-receptor antagonists (like Siponimod), which show a shorter half-life compared to their predecessor and have a higher risk of rebound effects [18]. Among oral medications for MS, Dimethyl-Fumarate (DMF) is the most prescribed [19]. It is thought to activate the transcription of an antioxidant response [20] through the nuclear factor erythroid 2-related factor 2 (Nrf2) [21]; however, a Nrf2-independent mechanism reducing cytokine production in the microglia has also been proposed [22]. Of note, no EMA- or FDA-approved disease-modifying treatments (DMTs) target disease mechanisms beyond immunomodulation [23]. Thus, the door for new targets and therapeutic strategies is open to innovative inputs.

## 2. Viral Infections Related to MS Development

A pivotal role in expanding the possible approaches for DMTs against MS could be played by the recent studies on Epstein–Barr virus (EBV) infection and the incidence of MS, which is based on the assumption that viruses have a key role in the development of the disease. Bjornevik et al. [24] conducted a retrospective analysis on more than 10 million enrolled US military personnel and found a 32-fold increased risk of MS after infection with EBV [24]. This correlation was not found for other viral infections. Also, the augmentation of serum levels of neurofilament light chain increased only after EBV seroconversion: this prompted the authors of the study to suggest EBV as the leading cause of MS [24]. This result sparked a great debate, due to the extremely high prevalence of EBV infection, which is thought to occur in 95% of adults worldwide regardless of being symptomatic or not [25]. The EBV infection has therefore been deemed a required factor, but on its own, not sufficient for the development of MS [26]. The mechanism leading from EBV infection to MS still eludes the researchers, though numerous hypotheses have been formulated. Among those, one of the most detailed involves HLA-DRB1*15:01, an allele of the *HLA-DRB1* gene constituting one of the most prevalent genetic risk factors for MS, that has been found to act as a co-receptor for EBV, possibly facilitating B-cell infections [27] and increasing susceptibility to MS [28]. Other studies have tested the interaction between EBV and MS-predisposing genes in a broader manner, elucidating a distributed dysregulation of MS-associated EBV interactors at the CNS level and related disease-specific mechanisms, e.g., the ones related to EBV nuclear antigen 2 (EBNA-2) expression [29]. However, the links between EBV infection and MS insurgency should still be more thoroughly studied: few examples that need further research include the possible interactions between EBV and innate immunity mechanisms, which have often been underestimated [30], or the still unknown differences in MS incidence between being infected by EBV as an infant or as an adult [31]. Altogether, these recent evolutions involving viruses, or viral sequences, in MS etiology lay their foundation on solid bases, but they do call for a deeper understanding and thus leave room for other interpretations and more elucidated mechanisms to explain MS insurgency and, consequently, new therapeutic approaches to adopt.

## 3. The Emerging Role of Human Endogenous Retroviral Sequences (*HERVs*) in the Development of MS

In the last 40 years, a possible correlation between anti-retroviral drugs and better motor and neuronal functions of MS patients has come to the attention of researchers and clinical experts alike [32]. Notably, a reduced rate of MS has been documented among individuals with HIV [33], which may be attributed to the immunosuppressive effects of HIV [34] and/or the impact of antiretroviral drugs [33]. The conceivable rationale behind this protective effect of anti-retroviral drugs resides in a new possible molecular actor in the pathological mechanisms leading to MS: human endogenous retroviral sequences (*HERVs*) [35]. These nucleotide sequences, with a largely unknown role, account for approximately 8% of the human genome [35]. Although *HERVs* were once dismissed as “junk DNA”, our understanding of their potential functions in both normal and disease-related processes has expanded significantly [36]. Despite their substantial portion in our genome, most *HERVs* have been silenced because of mutations such as substitutions, insertions, deletions, and as a result of epigenetic changes, which are all vertically transmitted in the population [36]. This has not forbidden proteins expressed from *HERVs* from exerting physiological roles. More in detail, the most studied protein expressed from an *HERV*, syncytin, has a progressively understood role in human placenta development [37]. Syncytin-1, expressed from the *ERVW-1* envelope gene, elicits an indispensable role for placental integrity during its development [38], while also contributing to immunomodulatory activity during pregnancy [39]. Syncytin-2 is instead coded by the *ERVFRD-1* envelope gene, and its most important role probably resides in preventing the rejection of fetal cells by the mother’s immune system [39] thanks to its immunosuppressing domain (ISD) [40].

During the years, emerging evidence has accumulated for a possible role of *HERVs* in MS pathogenesis [41]. The *HERV-K* class of endogenous retroviral sequences has been proposed as a genetic risk factor for MS [42] or a possible interesting option for a biomarker [43], although it has been mainly associated with amyotrophic lateral sclerosis (ALS) [44] or with carcinogenesis [45]. However, it is the *HERV-W* class of endogenous retroviral sequences that constitutes the main investigative focus for a therapeutic strategy [46]. *HERV-Ws* have been linked to the increased prevalence of MS in women due to their presence on chromosome X [47]. Researchers discovered a wide variety of *HERV-W* (as well as *HERV-H*) mRNA transcripts located at chromosome 7 that were exclusively detected in MS lesions [48]. Also, HERV-W class polypeptides have been repeatedly found in the brain of MS patients [41], and the HERV-W env protein is present in typical MS lesions [49]. Although HERV-W env is better linked to MS pathogenesis, the gag-encoded proteins from *HERV-W* sequences were also found to be significantly increased in MS-induced demyelinated neurons [50]. From an autoimmune perspective, HERV-W env seems mainly to trigger the innate immune response by interacting with TLR4 receptors in the brain [51]. However, the negative impact of HERV-W env might be closely related to the formation of typical MS lesions by blocking remyelination mechanisms [52] or interfering with oligodendroglial differentiation [53]. Altogether, the noxious dual impact of HERV-W on myelin repair and white matter lesions may represent a decisive one in the pathogenesis of MS [54] but also an appealing one for innovative DMTs. Finally, the HERV hypothesis does not contradict, but instead complements, the involvement of EBV in MS etiology. *ERVFRD-1* gene expression positively correlates with an increase in EBV load [55], and EBV glycoprotein 350 (gp350) stimulates *ERVFRD-1* expression [56]; therefore, HERV-W env may represent the missing link between EBV infection and the insurgency of MS.

## 4. Closing in on the Possible Antiretroviral Therapy of Choice Against MS: NRTIs

From a clinical standpoint, the therapeutic effect of antiretroviral drugs against MS gained consideration with the first reported cases of HIV and MS co-morbidity, which showed a significant improvement of the MS symptoms when the anti-retroviral regimen started [32,57,58]. However, clinical trials utilizing antiretrovirals against MS [59] or targeting HERVs in MS patients [60] showed some limitations that we would like to discuss in more detail, as they could usefully narrow down the therapeutic possibilities. In the first case, the clinical trial explored the efficacy of Raltegravir, an integrase inhibitor, in MS [59]. The study failed to reach the clinical endpoints, i.e., preventing the progression of RRMS [59]. However, while integrase inhibitors inhibit viral replication, they may suffer from an important limitation because they are ineffective in interfering with *HERVs* already integrated into the genome [61]. The second clinical trial was instead testing Temelimab, a monoclonal antibody that targets pathogenic HERV-W env protein [60] without eliciting functional impairment of physiological syncytin [62]. Whilst showing promising preliminary evidence for neuroprotective results, this study also failed to reach its primary endpoint in eliciting a positive impact on acute inflammation [60]. Other clinical cases suggested more efficient therapeutic alternatives to target HERV-W env, such as Nucleoside-analog Reverse Transcriptase Inhibitors (NRTIs).

NRTIs are a well-characterized treatment option against HIV and hepatitis B, and elicit their therapeutic effect by interfering with the reverse transcriptase enzyme and the following conversion of RNA into DNA, which is a crucial part of viral infections [63]. In fact, the first FDA-approved medication against HIV, Zidovudine, is an NRTI [64]. A few features of these antiretroviral drugs make them advantageous to this day in the fight against HIV: not only are they able to permeate cellular membranes through simple diffusion thanks to their hydrophobic properties, but they also show a higher affinity for the viral reverse transcriptase than the human DNA polymerase, which reduces their toxicity to human cells [63]. In addition, NRTIs have also proven their efficacy in inhibiting cytoplasmic reverse transcription of *HERVs* and counteract inflammation in neurological diseases [65], thus showing potential as a suitable option against MS due to the pro-inflammatory mechanisms of the disease and their possible link with endogenous retroviruses.

The first clinical case discussed in this review features a patient who was first diagnosed with MS and later with HIV. After HIV diagnosis, the patient began highly active antiretroviral therapy (HAART), following unsuccessful treatment with interferons for MS [32]. The drug combination involved a series of antiretrovirals, and since the introduction of this therapeutic regimen, his MS symptoms improved gradually; however, the only drug present from the beginning was Lamivudine (NRTI) and since the introduction of Abacavir (NRTI) the patient showed no relapses or MS-like symptoms at all, with the researchers readily hypothesizing that HAART could be beneficial for MS patients [32]. Another case of co-morbidity was reported regarding a patient who was examined after exhibiting a series of MS-like symptoms, and was found to be affected by both MS and HIV [57]; however, while being treated with corticosteroids for the acute exacerbations, the clinicians reported a remarkable improvement of symptoms possibly due to the antiretrovirals involved and, among these, the combination of two NRTIs, Emicitrabine and Tenofovir [57]. In the same year, a similar discovery of co-morbidity from MS and HIV was reported, regarding a patient being examined after manifesting visual impairments possibly related to MS [58]. After just two months of Glatiramer acetate treatment, this patient continued only with an HAART regimen. Remarkably, one of the two drugs associated with impressive recovery was once again the NRTI combination of Emicitrabine and Tenofovir [58].

A more recent case report provided an even more appealing development when exclusive treatment with Combivir, a combination of the NRTIs Lamivudine and Zidovudine, dramatically improved MS symptoms as well as reduced the typical histological MS lesion in an HIV-negative patient [66]. This particular occurrence happened because this patient, a medical student, read previous case reports on the beneficial effects of HAART for MS patients and, after being treated with Glatiramer acetate to no avail, independently started therapy with Combivir to success, which was claimed as a link to Zidovudine-related impairment of EBV replication [66]. A few years later, the first case involving no DMT for MS was reported when a patient diagnosed first with MS and then HIV was treated only with HAART because he was lost for follow-up between the two diagnoses [67]. The patient was treated, particularly, with the NRTI combination of Emicitrabine and Tenofovir as the one drug present for the entirety of his therapeutic regimen and showed no relapses, worsening mobility impairments or further histological lesions after his neurological follow-up resumed [67].

Finally, two reports from 2024 hinted at the beneficial role of Tenofovir for MS patients due to its role in hindering EBV replication and started clearly distinguishing between different Tenofovir formulations [68,69]; furthermore, they linked this clinically relevant effect to the recently strengthened evidence about EBV infection and its role in MS development [24]. The first one saw a patient first diagnosed with MS and treated with scarce success with DMTs for the worsening neurological conditions. The patient discontinued Fingolimod treatment, was subsequently diagnosed with HIV, and began HAART right away. Regarding MS, HAART resulted in clinical and radiological stability for the entirety of the treatment, even when lightly modifying the drug combination, but always adhering to Emicitrabine and different formulations of Tenofovir [68]. The second report described instead a patient who was first diagnosed with MS and then, after a successive diagnosis of HIV, stopped treatment with Fingolimod due to fear of rebounds and was treated with Tenofovir Alafenamide (TAF) towards a complete remission of the MS symptoms. After switching to the Tenofovir Disoproxil Fumarate (TDF), a less potent inhibitor of EBV replication, the patient showed one lesion but no clinical activity [69]. However, after a switch for convenience in the HAART treatment with Dolutegravir (integrase inhibitor), Rilpivirin (a Non-Nucleoside-analog Reverse Transcriptase Inhibitor, NNRTI) and no NRTIs, more than one MS lesion and a clinical relapse were reported [69]. Altogether, these clinical trials and case reports show a clear pattern for clinical improvement or stability of symptoms in the presence of NRTIs and worsening conditions or inability to stop disease progression in the absence of NRTIs. The findings of the reported clinical trials and cases are summarized in Table 1:

## 5. Conclusions: NRTIs as Suitable Option for MS Treatment and Future Perspectives

This review offers a renewed insight into a rising topic among MS therapeutic options. The recent light shed on the role of viral infections in the development of the disease is a refreshing element for the hopes of MS patients, but it certainly needs refinement. The rationale behind the proposed implementation of NRTIs against MS could indeed not only be clinically solid, according to the growing evidence here reported, but also fill the gaps between the recent discoveries about EBV infection and the etiology of the disease. What is more, the hypothesized beneficial effects of NRTIs should not be limited to their hindering of EBV replication, as *HERVs*, and especially HERV-W env, offer many new possibilities for targets that deserve a deeper understanding, especially because it could provide new DMTs that finally allow remyelination and tackle the disease from another perspective: in vivo transgenic models suggest an important role of HERV-W env in enhancing the degeneration of myelin sheath due to the induction of a complex neurodegenerative environment [54]; in contrast, in vitro tests have shown that blocking the negative effect on remyelination by HERV-W env can lead to a full rescue of this dysregulation [53]. Also, NRTIs by themselves showed potential against neurodegenerative diseases; particularly, a potential beneficial effect against Alzheimer’s Disease was proposed, due to NRTIs (e.g., Lamivudine [65]) blocking NRLP3 inflammasome activation [70]. The neurodegenerative, inflammatory characteristics of MS can therefore be targeted by NRTIs, and this rationale further calls for deeper inspection due to the possible pathogenic role of *HERVs* in MS development. Retrotransposition of endogenous elements can lead to RNA/DNA hybrids that have been shown to activate NRLP3 inflammasome [71]; therefore, the aforementioned benefit from NRTIs against this specific pathogenic mechanism could prove instrumental also in treating MS. Altogether, the mechanisms behind this observed specific beneficial pattern of NRTIs against HERV-W env (and, consequently, against MS) show promise, but need further studies to be truly understood in their complexity; however, the therapeutic superiority of NRTIs reported in this review, when compared to other classes of antiretroviral drugs, should not be overlooked, and instead be thoroughly tested from both a clinical and a biological point of view.

The first and probably most immediate of the consequences of the rationale presented here is surely the repurposing of NRTIs against MS. Already licensed drugs for other diseases, such as Tenofovir, Emicitrabine or Combivir, could be tested for their efficacy to stop typical MS lesions and the progression of the symptoms in large-scale clinical trials. In Australia, an association of scientists, the Australian Anti-EBV Drugs for MS Working Group, has screened 11 drugs with anti-EBV activity and shortlisted four of those as the ones needing most immediate evaluation through Phase III clinical trials [72]. Among those, Tenofovir, particularly the TAF formulation, was present, and currently, to our knowledge, three clinical trials involving three of the four selected drugs are underway, investigating whether these compounds can improve MS outcomes by suppressing EBV activity [73]. Going more into detail about the possible utilization of NRTIs against MS, the FIRMS-EBV clinical trial (registration number: ACTRN12624000423516) is a randomized, placebo-controlled Phase III study that follows the screening from the Australian Anti-EBV Drugs for MS Working Group and is testing whether TAF (or Spironolactone) can reduce fatigue in RRMS patients [74]. Also, a Phase II interventional study (ClinicalTrials.gov ID: NCT05957913) held at the Massachusetts General Hospital is testing if the combination of Emicitrabine and TDF (commercially available as Truvada) is able to reduce EBV levels in blood and saliva and, also, if it is safe and tolerable for MS patients [75]. However, it is important to remember that said EBV activity could not be the end of the story for MS development, and underline again that *HERVs*, particularly HERV-W env, offer a target that could potentially lead to remyelination [54] and also avoid worsening the effects of viral infection. Therefore, repurposing of the other NRTI drugs could very well open a plethora of new chances for clinical improvement of MS patients.

Perhaps even more intriguing is the possibility that existing, licensed NRTIs could offer the basis for new and improved drugs, counting on growing knowledge about not only their structural features, but the ones of *HERVs* as well. As an example, a very recent study explored the structural conservation of the various HERV domains and sequences in our genome, and a few conserved features across the *HERV* families were observed [76]. While it remains unclear if it happened due to regulatory or structural reasons, this genome-wide map of conserved *HERV* features could narrow the target for therapeutic approaches, possibly aiming at conserved domains for structural reasons or finding out which ones are present only in pathogenic situations to select them for new therapies. As an example, HERV-K reverse transcriptase and HIV reverse transcriptase showed some significant structural resemblances [77], thus concretely opening the door for further functional refinement of NRTIs to target HERV sites that can be conserved among different families and remain susceptible to NRTI therapeutic action. The knowledge regarding structural features of HERV-W env or envelope proteins from other *HERV* families is growing as well [78,79], thus providing new possibilities to narrow down the important features of *HERVs*, and their envelope proteins, for the insurgency of MS.

In conclusion, a growing body of evidence for the implementation of NRTIs in MS therapeutic strategies warrants for more investigation about the topic, and offers new possibilities on repurposing existing drugs or adapting them into improved molecules to target MS. This approach could also open a new era of DMTs that would not only focus on immunomodulation, but also on myelin sheath repair, focusing on the most fundamental pathogenic traits of MS and offering a new hope for MS patients.

## Figures and Tables

**Table 1:** Clinical case reports and relevant trials linking NRTI-containing antiretroviral regimens (and non-NRTI interventions) to clinical outcomes in multiple sclerosis.

Clinical Trial/Case	Study/Patient Context	NRTI Regimen	Clinical Outcome	Notes
Gold et al. [59]	INSPIRE phase II trial: Raltegravir (integrase inhibitor) in RRMS.	No NRTI used (integrase inhibitor only).	Failed to reach clinical endpoints (no prevention of RRMS progression).	Integrase inhibitors block viral replication but do not affect *HERVs* already integrated into the genome [61].
Hartung et al. [60]	Randomized phase 2b trial of Temelimab (anti-HERV-W env mAb).	No NRTI used (monoclonal antibody only).	Promising neuroprotective signals but failed primary endpoint on acute inflammation.	Temelimab targets HERV-W env without impairing physiological syncytin; did not show benefit on acute inflammatory endpoints.
Maruszak et al. [32]	MS diagnosed first, then HIV; prior interferon failure.	Lamivudine initially; Abacavir added later.	No relapses; sustained clinical stability.	Authors hypothesize HAART benefit; effect attributed to NRTIs.
Delgado et al. [57]	MS and HIV; treated with corticosteroids for exacerbations.	Emtricitabine + Tenofovir.	Marked clinical improvement.	Ambulation did not require any assistance after HAART started.
Chalkley et al. [58]	MS and HIV; Glatiramer acetate for 2 months, then HAART only.	Emtricitabine + Tenofovir.	Marked recovery; maintained on HAART.	Improvement occurred after switching to HAART alone.
Drosu et al. [66]	HIV-negative patient self-initiated therapy after Glatiramer failure.	Combivir (Lamivudine + Zidovudine).	Dramatic symptom improvement; reduced histological lesions.	HIV-negative patient; authors suggest Zidovudine may impair EBV replication.
Labella et al. [67]	MS, then HIV; lost to neurological follow-up and treated only with HAART after HIV diagnosis	Emtricitabine + Tenofovir	No relapses; no radiological worsening on follow-up	First reported case with no concurrent DMT and sustained stability.
Drosu et al. [68]	MS with poor DMT response; later diagnosed with HIV and started HAART	Emtricitabine + various Tenofovir formulations	Clinical and radiological stability during HAART	Authors link Tenofovir to EBV inhibition; minor regimen changes did not alter outcome.
Torkildsen et al. [69]	MS, then HIV; stopped Fingolimod, started TAF → TDF → later switched to non-NRTI regimen	TAF → TDF; later regimen without NRTIs	Complete remission with TAF; one lesion with TDF but no clinical activity; one relapse and multiple lesions after switching to regimen without NRTIs	Pattern suggests disease control associated with presence of NRTIs.

## Data Availability

No new data were created or analyzed in this study.

## Abbreviations

The following abbreviations are used in this manuscript:

NRTI	Nucleoside-analog Reverse-Transcriptase Inhibitor
MS	Multiple Sclerosis
EBV	Epstein–Barr Virus
HERV	Human Endogenous Retroviral sequences
HERV-W env	HERV-W envelope
CNS	Central nervous system
IFN	Interferon
RRMS	Relapse-remitting MS
PPMS	Primary progressive MS
SPMS	Secondary progressive MS
S1P1	Sphingosine-1-Phosphate-1
DMF	Dimethyl-Fumarate
Nrf2	Nuclear (erythroid-derived 2) related factor
EMA	European Medicines Agency
FDA	Food and Drug Administration
DMT	Disease-modifying treatment
EBNA-2	EBV nuclear antigen 2
ISD	Immunosuppressing domain
ALS	Amyotrophic lateral sclerosis
TLR4	Toll-like receptor 4
HIV	Human immunodeficiency virus
HAART	Highly active antiretroviral therapy
TAF	Tenofovir Alafenamide
TDF	Tenofovir Disoproxil Fumarate
NNRTI	Non-Nucleoside-analog Reverse Transcriptase Inhibitor
gp350	EBV glycoprotein
NLRP3	NLR family pyrin domain-containing 3
